# Exploring the Experiences of LTCF Staff in Implementing Visitation Policies in England During the COVID-19 Pandemic

**DOI:** 10.3390/ijerph22020221

**Published:** 2025-02-05

**Authors:** Danni Collingridge Moore, Natalie Cotterell

**Affiliations:** Division of Health Research, Lancaster University, Lancaster LA1 4YW, UK; n.cotterell@lancaster.ac.uk

**Keywords:** coronavirus, COVID-19, long-term care facilities, care homes, nursing homes, health policy, public health, social care

## Abstract

**Background:** Restrictions on family visitation to long-term care facilities (LTCFs) during the COVID-19 pandemic remain an area of contention for residents, family members and staff members. Current research has explored the experience of family members; however, fewer studies have explored the impact of visitation restrictions from the perspective of LTCF staff members. We examined the experiences of LTCF staff members in implementing visitation restrictions, including maintaining contact with families, in England over the course of the COVID-19 pandemic. **Method**: A sample of twenty-four LTCF staff members employed at eight LTCFs in one region of England was recruited. Qualitative, in-depth interviews were conducted with staff members to explore their experiences of implementing government policies during the COVID-19 pandemic. Thematic analysis was used to analyse data on maintaining contact and visitation with relatives. **Results:** Five broad themes were identified from the data. These were: (i) engaging with family members on visitation, (ii) facilitating visitation with family members, (iii) maintaining remote contact with family members, (iv) managing visitation restrictions with residents and (v) navigating equitable access for residents and family members. While some staff welcomed the introduction of national guidance on restrictions as a source to refer family members to for justification of the LTCFs’ decisions to restrict access, others reflected on the negative effect of limited social contact on resident wellbeing and difficulties in implementing the guidance. At times, LTCFs felt conflicted in their responsibility for supporting family members in visiting at the same time as communicating, enforcing and policing visitation restrictions. Guidance on facilitating remote contact required substantial time and resources required to support it. **Discussion:** The extent to which restricting visitation was a proportionate response to reducing the spread of COVID-19, within the wider context of negative impacts on relatives and family members, is an ongoing debate. This study identified some of the challenges experiences by LTCF staff in implementing such guidance, and calls into question the practicality of restricting visitation. Further research is needed on how social contact can be maintained between relatives and their families during pandemics, which is both equitable and achievable.

## 1. Introduction

Globally, long-term care facilities (LTCFs) for older adults were one of the hardest-hit groups within society by the COVID-19 pandemic. It is estimated that around 40% of the total COVID-19 deaths across Organization for Economic Co-operation and Development (OECD) member countries came from the long-term care sector [1]. In England alone, age-standardised mortality rates were approximately ten times higher among LTCF residents compared to those among older adults residing in the community during the first wave of the pandemic [2]. Similar trends have been identified in countries with comparable ageing populations and long-term care systems. In Canada, for example, the first year of the pandemic resulted in over 14,000 resident deaths, representing more than two-thirds of the country’s overall COVID-19 deaths [3].

The nature of the long-term care system for older adults in England has a number of characteristics which made it more vulnerable to the effects of the pandemic. Older adults residing in LTCFs are usually in relatively poor health; in 2021, 56.4% of residents were aged 85 years and over, 31.8% rated their overall health as bad or very bad and 70.9% self-reported that they were limited a lot in their ability to go about day-to-day activities [4]. The physical environment within LTCFs often comprises multiple communal settings, such as meal areas, common rooms and shared bathrooms, resulting in frequent contact between staff members and residents, with some studies estimating that resident-to-staff contact occurs around 5.8 times per hour [5]. Thirdly, transfers between health care settings can be high, such as admission to and discharge from hospital, with frequent visitation from wider health professionals, in addition to loved ones [6,7].

Internationally, restricting visitation to LTCFs was one of a range of policy responses put into place to reduce COVID-19 transmission within the long-term care sector. Many countries either banned visits altogether or implemented some kind of restriction such as: increased use of personal protective equipment (PPE); social distancing between residents and between staff members; and/or mandatory testing of visitors prior to entry [1,8]. In England, the stringency of visitation restrictions varied from no visits being allowed during periods of lockdown, with the exception of visits to residents who were approaching end of life, increasing to one consistent visitor in July 2020, two constant visitors in April 2021 and five visitors in May 2021, with restrictions on the number of visitors finally lifted in July 2021 [9]. During the time periods when visits were advised or permitted, additional restrictions were put in place, such as limiting visitors to one designated and constant individual or to one room, minimising contact with other residents and staff members and keeping personal interaction between the family member and resident, including physical contact, to a minimum [10,11]. The guidance was constantly being updated and changed, often with little notice provided to LTCF staff, in response to developments in COVID-19 transmission across the country, and in the later stages of the pandemic these changes were dependant on the rates of infection within the local authority the LTCF was allocated to [12]. The guidance also proposed using remote contacting methods for LTCFs to maintain contact with relatives, such as using telephone or video-calling, and emphasised the need for regular, personalised updates on residents [13]. Funding streams were provided to support visitation, which could be used to backfill the additional staff time required to facilitate visits, such as providing testing for visitors, or to create safe spaces for visits, such as visitation pods or specific rooms with additional infection control policies [14].

Reducing the number of interactions between residents and visitors within a LTCF would likely have t reduced the transmission of COVID-19 infection; however, the extent to which the negative, knock-on effects of visitation restrictions outweighed the benefits, and to whom, is debatable. For residents, reductions in social interactions due to the protective measures introduced during the COVID-19 pandemic have been associated with a detrimental impact on both residents’ and their relatives’ wellbeing [15,16]. In one study, rates of depression and the incidence of substantial unplanned weight loss, cognitive functioning and incontinence among nursing home residents worsened after the onset of the pandemic and associated restrictions [17].

For relatives, the emotional impact of being unable to visit their family member residing in the LTCF, or visiting with additional restrictions, was also challenging. Family members reported feeling a sense of anger, frustration and guilt at not being able to visit their loved one, alongside fears that the resident may feel they had been abandoned [18,19,20]. In addition, reductions in onsite inspections by the Care Quality Commission (CQC) meant that there was less oversight into the quality of care being provided within LTCFs, as few people external to regular staff members were able to access the facility [21].

The experiences of LTCF staff members who were administering the visitation restrictions put in place during the COVID-19 pandemic are less understood. The aim of this study was to explore the experience of LTCF staff members in maintaining contact with families, including facilitating visitation, in England during the COVID-19 pandemic. In doing so, it explores the barriers and challenges to managing the relationships between relatives, residents and staff members in the context of implementing visitation restrictions and identifies areas for further research and policy development to inform future guidance on this area during future pandemics.

## 2. Methods

### 2.1. Methodological Approach

In England, LTCFs are registered as either providing “residential” care, where onsite care is provided by health care assistants, and nurse/physician care is provided externally through the National Health Service (NHS), or “nursing” care, where onsite care is provided by health care assistants and nurses, with physician care provided externally through the NHS [22]. Over 85% of LTCFs in England are operated by for-profit providers [23]. It is estimated that 58–66% of LTCF staff are employed on a full time basis, compared to 42–34% part time staff [24]. Prior to the pandemic, the median annual turnover of staff level in LTCFs was 22.7% of total staff annually [25]. LTCFs providing nursing care have significantly higher staff vacancy rates, staff turnover, number of contract staff and a significantly lower staff retention rate [26].

In the original study, LTCFs were recruited as case studies to explore the experiences of LTCF staff in implementing policy recommendations on the management of COVID-19 in such settings. This paper reports the secondary analysis of data collected during semi-structured interviews with staff members within the case studies, focusing specifically on the theme of visitation. Twenty-four participants were recruited from eight LTCFs in the north-west of England (see Table 1 for full characteristics).

LTCFs were informed of this study through the Enabling Research in Care Homes Network, who advertised this research. The inclusion criteria for the participation of LTCFs in this study included registration with the CQC to provide care to older adults, being active during the COVID-19 pandemic and rated as either outstanding, good or requiring improvement at their most recent CQC inspection. LTCFs that were rated as inadequate (the service was performing below standard and action was being taken against the person or organisation that runs it) were excluded from taking part in this study, on the basis that placing additional time and resource pressures on staff in a facility that was struggling to provide adequate care to residents would be unethical. Staff members were eligible for participation if they were aged 18 years or over, were able to participate in the interview in English and worked at the LTCF during the COVID-19 pandemic.

Semi-structured interviews were conducted in October 2023–March 2024, either online using Microsoft Teams or in person (at the LTCF) and were between 40–60 min long. All interviews were audio recorded, transcribed using Microsoft Teams, and the interview transcripts and the associated data were managed, organised and stored using Atlas.ti. software 24 [27]. The interview transcripts were analysed using thematic analysis, which consists of five steps: data familiarisation, generation of initial codes, searching for themes, review of themes and, finally, defining and naming the themes [28]. Study reporting was informed by the COREQ checklist for the comprehensive reporting of qualitative studies [29].

### 2.2. Ethics

The managers of all LTCFs included in this study provided written informed consent. All staff who took part in an interview provided written informed consent to take part and could withdraw their consent at any time. All names have been changed to pseudonyms to protect anonymity and identifying characteristics were removed where necessary. The Lancaster University Faculty of Health and Medicine Research Ethics Committee provided ethical approval (reference: FHM-2023-3368-RECR-3).

## 3. Results

Eight LTCFs were recruited to this study; seven were privately owned and one was not for-profit. Four provided residential care only, four provided nursing care and three had a specialist unit for dementia care; LTCF size ranged from 25 to 120 beds. Interviews were conducted with 24 LTCF staff members, the majority of whom were female (n = 21) with ages ranging from 23 to 68 years old (mean = 46). The mean number of years participants spent working in direct patient care was 21 years (range 5–46), with the mean number of years spent working in LTCFs being 18 years (range 5–46). The majority of staff who participated in the interviews worked full-time (79.2%).

Five broad themes were identified from the data. These were: (i) engaging with family members on visitation, (ii) facilitating visitation with family members, (iii) maintaining remote contact with family members, (iv) managing visitation restrictions with residents and (v) navigating equitable access for residents and family members.

### 3.1. Engaging with Family Members on Visitation

The first theme that emerged from the data related to staff members’ experience of the challenges encountered communicating and enforcing restrictions on visitation with family members. LTCF staff understood and could empathise with the feelings of family members, especially those who felt angry, frustrated and guilty that they could not physically visit their relatives within the LTCF. Participant 015 discussed the effect it had on them as a staff member, and being a bystander to this suffering:

“*I think it was a lot harder for families, the families themselves must have felt awful, you know, so guilty and whatever that they couldn’t touch or hold them or whatever, because that’s what a lot of them do when they come here, they’ll just sit and you know, hold them or whatever, but they couldn’t do any of that, and that was the hardest part, seeing families […] I mean, we kept saying phone up, you know, we’ll talk to you as much as we can on the phone and let you know what’s happening, but it was worrying for them*.”

P015, F, Housekeeper

In some cases, this could be reflecting on the reaction of family members being told that they were not allowed to visit the facility; in others, it related to the challenges of facilitating visits where family members were unable to engage in close or physical contact with relatives due to social distancing restrictions in place during visits. Witnessing such interactions, at times, left LTCF staff members with the view that the choice of whether to visit should have been the responsibility of family members and residents to make, rather than down to the LTCF, local government or national guidance:

“*So yeah, it wasn’t a great time and it was horrible knowing that, you know, families couldn’t come in. We had poorly residents that we kind of knew that had it, they probably never got tested for it and that, you know, families had to watch their loved ones from a window from outside if they were lucky, you know, because if they were upstairs, we couldn’t get a ladder up and do it that way, so yeah, it was so horrible when it was all the time. Even thinking back now, it was like it never happened, but it did happen*.”

P008, F, Housekeeper

In general, staff members felt that the majority of families were understanding of the restrictions that were put in place by the LTCF management and recognised the role of these restrictions in protecting their loved one and other residents within the facility:

“*But the families were very supportive. I only had one issue with one family member who thought it was a right to see her dad and all the rest of it and those big battles went on and she was moving him, yep jog on, move him […] You know the other 99.9% of families were all really, really supportive. Everybody was, which made it so much easier for me as well.*”

P002, F, Registered manager

“*I’ve also seen some of the families who are not even bothered in that sense, because they didn’t want to spread their infection to their loved ones. They would rather stay away and, you know, make their loved ones safe*.”

P010, F, Clinical manager

The publication of government guidance provided a legislative reference for LTCF managers to refer to in justifying the restrictions that had been put in place. In the early stages of the pandemic, prior to national guidance on restrictions in adult social care settings being developed, some LTCFs that were pre-emptively limiting visitation welcomed publicly issued government guidance and viewed it as overdue given the clear potential for the increased risk of COVID-19 infections for both residents and staff members within long-term care settings:

“*I can remember, I was at work, it must have been on the late (shift) when Boris Johnson announced that there’s going to be the lockdown and I can remember we were all absolutely overjoyed, we were so excited and thrilled that he’d actually done this because it was just like, it was scary and something needed to happen and we felt it should have happened sooner really.*”

P021, F, Care manager

As the pandemic progressed, the guidance was especially useful when working with family members who were either unhappy with the restrictions or questioned their validity. Participant P017 reflected on using the guidance as a shield when the facility approach to restricting visitation was questioned by relatives who were unhappy:

“*Visiting wise, a few families were very, very upset and angry, there was one family in particular, and the relative got really angry at not being able to be allowed to come in, not being able to visit and we just kept pushing, pointing the government guidance out and sending it out and saying look, we’re just having to follow this at the end of the day. They weren’t happy*.”

P017, F, Senior care assistant

Throughout the frequently updated guidance on visitation restrictions, a central issue that emerged across the participants interviewed was managing the dual role of both facilitating and supporting visitation, at the same time as communicating the current regulations to visitors and enforcing the associated restrictions:

“*We did have a complaint which said we were breaching human rights and, you know, her family member is a barrister and really took us to task. At the time I was, there wasn’t a director, so I was dealing with that complaint as deputy and that was quite stressful because obviously you don’t want to be breaching someone’s human rights, but equally you’ve got to think there’s 40 people living in that service and you know, without it being like that herd mentality, it was trying to kind of look after everybody*.”

P018, F, Head of operations

The dichotomy was apparent throughout interactions with family members, both prior to lockdown being announced and in the coming months. In particular, LTCF staff felt responsible for making decisions on visitation policies which balanced the needs of an individual resident with protecting the health of all residents in the facility, which at times created a divide between staff members and family members:

“*There was only one family, who came in, and then he rang up and he said, ’oh, I have been for a COVID test and I’m positive I thought I was but… and I thought, well, why did you come in the home? Why did you bring it in the home? Why did you bring it to your mother?*”

P006, F, Registered manager

LTCF staff were ultimately responsible for enforcing restrictions, such as limiting the number of visitors, denying visits from relatives who were viewed as having a higher risk of spreading COVID-19 infection, such as key workers and those who visited when experiencing COVID-19 symptoms but chose not to test prior to the visit, in addition to responding to relatives’ complaints.

### 3.2. Facilitating Visitation with Family Members

The second theme, facilitating visitation, related to LTCF staff reflecting on the often novel approaches to ensuring visits were able to occur within the limitations specified in the government guidance issued at the time. In some cases, this would include using outside seating areas, preparing visiting pods, using plastic screens or limiting visits to specific areas (or the rooms of individual residents).

“*I mean, we have the conservatory down there, you know, facilitated visits after we screened them and things like that. That could have happened from the beginning, you know those screens could have gone up. Families could have come and visited, you know?*”

P011, F, Deputy care manager

“*We built a pod, quite massive really so all the families could come round the side of the building right and through into the pod […]. We employed two girls as what we call visitor coordinators, so they were in charge of organizing the visiting slots and cleaning the pod after every visit, skyping, you know and we had the iPad […] But you see, some were bed bound you see, some couldn’t get down the stairs and, plus if they had a dementia diagnosis or something like that, they didn’t understand, you know? So in that case, we just put the phone that the family could talk and, you know, just hope that they could hear their voice, you know. It was difficult*.”

P006, F, Registered manager

Further to creating or utilising specific areas within the LTCF, facilitating visitation placed additional pressures on staff time. This included time to prepare appointment systems, administer tests to visitors and log the results, support visitors in putting on and taking off PPE and provide additional infection prevention and control (IPC) measures, such as disinfecting areas designated specifically for visitation. These tasks were in addition to providing care to residents, and combined with wider staff shortages, assigning roles to support visitation was challenging, and limited the staff time available to provide day-to-day care for other residents.

“*For us, the, visiting was the biggest challenge because of the time consuming, because of the limited numbers, the appointment system, the testing of the visitors, the PPE making them wear the PPE, I would definitely say out of that it was the visitation […] and having to be in separate rooms, so you’d have to have time slots cleaning the rooms in between. Everything about it was just extra work.*”

P014, F, Deputy care manager

The dual role of simultaneously facilitating and supporting visitation, while also enforcing restrictions, extended to engaging with relatives at times when visitation was allowed. In these cases, the role of LTCF staff members was both to support visitation, through the tasks discussed previously. Within the same interaction, LTCF staff were both expected and required to ensure family members were limited to one, two or five visitors (depending on when the guidance was issued/updated), enforce restrictions on physical contact between the family member and the resident and limit the length of time that the visit lasted. The extent to which family members who did visit acted within the issued guidance varied, with some subverting the rules to ensure greater access. There was also a sense that LTCF staff felt that some of the restrictions were unrealistic for families to adhere to, such as restricting physical contact between family members and relatives. Often, care staff viewed physical contact as important and found it difficult to ask visitors to refrain from not maintaining social distancing. In cases where family members were actively breaking the rules, such as sneaking additional visitors into the facility or removing their masks, it was especially difficult for staff members to intervene. This was compounded by an underlying sense of empathy and understanding from staff members, some of which reflected on their own motivations and needs if they were in a similar situation with their own family.

“*Could only have so many visitors at a time, all that nonsense. One visitor would come in to see his mum. He’d opened the window and his sister would climb in the window. Yeah, absolutely crazy. And we don’t know how long that had been going on for. It’s just that somebody had gone in to give her a drink or something like that, and was like—how? There’s two of you in here, and she’d climbed through the window*.”

P014, F, Deputy care manager

“*It was difficult. It was, you know, you just felt for them. You felt for everybody you know. And like I said, being in that situation with my father, it made me, not to sound stupid, but it made me fight more for the residents and the families because, it’s changing the subject a bit, but this is how I feel because he (father) was receiving home care and they sent him into hospital. I wasn’t allowed to go, and then I had a choice again—Do I go into a hospital which is full of COVID and bring it back here or do I…?*”

P006, F, Registered manager

Again, such situations put LTCF staff in a difficult situation, assessing the risk of either allowing extra access or restricting further visitation and balancing the relationship with family members against the potential risks to the resident being visited, wider residents and staff members.

### 3.3. Maintaining Remote Contact with Family Members

The third theme which emerged from the data, maintaining remote contact with relatives, can be separated into two subthemes, facilitating remote contact and social media use. In addition to restrictions on visitation, the guidance supported alternative options to maintain social contact for residents, such as using telephones or video-calling, where possible.

“*There’s quite a few that, you build up, that like we become their family and they’re like… so a lot of people I know that, we’re not supposed to but I said I’ll just WhatsApp you if you don’t call when I come in, it’s the least I can do, you can’t see your families. So I just use mine or I bring my iPad in and just do a teams with them or a WhatsApp video call thing just so they can see them… So at least you had that. Yeah […] I know we’re not supposed to, but that’s what I would want if it was my grandma*”

P001, F, CHAPs team leader

Implementing remote contact between residents and their families required additional time and resources, such as staff time to coordinate and support residents in using tablets and mobile phones, sitting with residents who were unable to hold or use the technology without support, and the additional cleaning required to maintain IPC and PPE standards when supporting more than one resident.

“*But then at the same time it was, you know, families were ringing up as well and they wanted to see the families, so we do like video calls and stuff, but even then going in a room, it was like we had to take all the PPE off, we had to clean the phone that they were touching, it was everything, everything that we had to do, like door handles, key pads, pens…”*

P008, F, Housekeeper

In terms of timing, staff were not always available to support remote contact when residents or families wanted to call or at short notice, due to the time commitment required against providing general care to multiple residents. In the early stages of the pandemic, this additional workload was compounded by staff shortages, increases in administrative tasks (such as the reporting of COVID-19 cases) and caring for residents with increased care needs due to COVID-19 infection.

“*What I’m saying is you can’t social distance when you’re doing a job like this, and what was hard as well. I mean, we’ve got some residents who do like a cuddle and it’s like I can’t cuddle you, I cant […] I mean, obviously when we used to turn (them) we’d kind of like get a sneaky one in. […]. And that was the hardest bit really, cause to some of them we are all they see, some of them. Yeah, because they don’t have, some of them, don’t have families and that, and they are like family, they mean a lot to us*.”

P020, F, Senior care assistant

Staff also questioned the extent to which remote contact was equivalent in meaning and emotional/social engagement compared to face-to-face contact, especially for residents with dementia.

“*The family sometimes called doing a video call, but it’s not the same because they’ve got dementia, you know. They don’t know how to hold a tablet and talk with them. They were a bit frustrated with the family … they cant have a real conversation with the relative because it’s not the same, when you can give a hug, And then sometimes, when they come with the face mask, they don’t recognise because they have a facemask,, they just have to, like quickly say, ’mum it’s me’. That was a bit hard as well*.”

P016, F, Care assistant

Understandably, family members were keen to stay informed on the health and wellbeing of residents within the LTCF, and to be updated on any COVID-19 outbreaks and changes to visitation restrictions as soon as possible. In the context of the time and resource limitations related to understaffing and increased workload, some LTCFs discussed the potential of using social media to update multiple relatives at the same time, such as setting up Facebook groups and Whatsapp group chats for relatives. In doing so, this allowed mass communication during periods when administrative staff time was in short supply and reduced the demand from relatives for staff to facilitate time consuming, one-on-one contact with individual relatives. By posting photographs and videos of residents and their daily lives, such social media allowed relatives to see what life was like within the LTCF during lockdown, which it was hoped would provide reassurance regarding the quality of care being provided.

“*So we said, you know, FaceTime anything like that, we would do our best to support, and then the other thing that was thought, which is actually a really good thing, we continued, was we did our own Facebook group and we put a lot of pictures on, videos so people could see them, if they weren’t, like doing the virtual meetings, they could see what was going on and we’re really upped what we did in terms of the entertainment*.”

P017, F, Senior care assistant

However, these platforms also had the potential to unite relatives against the LTCF; in one case when a LTCF notified relatives of a COVID-19 outbreak on social media, the platform was used by relatives to post negative comments related to the quality of care provided by staff members, resulting in the closure of the social media site.

“*Yeah, well, they didn’t understand how hard it was, so we took it upon ourselves. The quickest way to get out to the relatives, because we had no admin at the time, the quickest way for us to get it out was to post on Facebook that we have got COVID and these are the options that we have, because obviously we had no admin staff, the manager was on the floor. There was no other way of doing it, and the comments we got on there, we had to shut the Facebook page down*.”

P013, M, Activity coordinator

Since visitation restrictions were removed, the use of both remote contacting and social media has continued to be integrated within some of the LTCFs that participated in this study, allowing families who live locally and further afield to stay informed. This approach allows relatives who lived abroad to remain in contact with residents, which in some cases was not in practice prior to visiting restrictions being put in place.

### 3.4. Managing Visitation Restrictions with Residents

A fourth theme related to managing the impact of visitation restrictions on residents within the LTCF. In addition to navigating relationships with relatives within the context of visiting restrictions, the relationship between LTCF staff and residents was impacted. Explaining the restrictions on visitation, and the implications of the pandemic in general, was problematic for staff members, both in terms of why the restrictions were in place, and what this would mean in practice for the residents. Residents, especially those with dementia, were often either unable to understand or enact social distancing measures, which was challenging for staff members to manage, at the same time as continuing to provide routine care. In addition, the requirements for staff to wear PPE, specifically masks, led to difficulties in residents recognising, understanding and communicating with staff, further exacerbating these challenges.

“*It’s hard to explain to them when they say, ‘why are you using this, why?’ And we say because of the pandemic, the virus is over the wall. They say ‘no, what are you saying’ you know. It was very difficult to explain to them*.”

P016, F, Care assistant

The negative effect of restricted visitation on the health and wellbeing of residents was apparent throughout the eight LTCFs involved in this study. Staff noticed a correlation between the onset of restrictions and a decline in some of the residents’ physical, mental and emotional health and in their engagement with staff, with other residents and in wider activities within the facility.

“*For the first year, nobody walked in there apart from paid staff, and actually that felt quite isolating for everybody, really unnatural*.”

P018, F, Head of operations

Part of this effect was attributed to changes in daily routine; as family members were no longer visiting, the incentive to prepare for visits, such as getting out of bed or getting dressed, were no longer required. In addition, activities such as eating meals, which may have previously been a focus of or associated with a visit from family members, were no longer occurring. The reduction in such tasks was seen as contributing to physical and emotional deterioration among residents, which was difficult for LTCF staff to witness.

“*Yeah, a lot of them went downhill, you know, not having family and stuff to talk to them. I’ve noticed that a lot of them went really depressed. Didn’t wanna get out of bed or anything anymore. They didn’t see the point in it. … a lot of them stopped eating and stuff. The family said just let us in and we can come in and feed them, but we couldn’t. And then when, like, if the rooms are downstairs, then we’ll let the family come to the bedroom window and whatever, and we had a pod after a bit that the people from upstairs could use. But, like the ones that couldn’t get out of bed, it weren’t really fair. They had no choice, so there was no way they could see their family*.”

P007, F, Senior care assistant

However, in some facilities, these changes in routine were associated with positive experiences, specifically related to the development of friendships between residents and improvements in the behaviours of residents with dementia. The routine of family visits, specifically the departure of family members at the end of the visit, which could trigger strong emotions in some residents, were removed.

“*Our actual residents were a lot easier and they were a lot more calm, and we could do more with, you know, sort of we had like a routine and we could keep to that routine for them, which made it a lot easier. When we’re like we are now and we’re back to normal. We’ve got residents, families coming in all the time. So we’re sort of, they’ll be sat downstairs chatting and all of a sudden the family will come. So we have to take them out of the room and take them upstairs there so they can have a visit. Some of them don’t want to move, they don’t want to visit, they don’t want to do this, that and the other, and when the families have been, they can be really unsettled for a few hours after and we didn’t have any of that. They all became quite close knit, you know, who sat where, they all had their own chair that they liked to sit or their own friends that they like to talk to […] so for a while it was quite nice, you know, a nice atmosphere in work because we were sort of sheltered. We were in our own little bubble*.”

P015, F, Housekeeper

Alternatively, under visitation restrictions, residents had more time and opportunities to interact with each other, allowing for friendships to develop between residents and contributing to a wider sense of community within the LTCF.

“*There was one lady who I was particularly worried about because her daughter used to visit a lot and she was always, ‘oh, I need to go to my room after lunch because my daughter will be in’ and I thought she’s really going to miss her. Do you know what? She blossomed throughout that which was really quite strange. She started interacting more with the other residents and built up little friendships*…”

P002, F, Registered manager

### 3.5. Navigating Equitable Access for Residents and Family Members

The fifth and final theme, the issue of navigating and ensuring equity between residents, relatives and staff members regarding social contact, was integrated within the previous themes discussed. The first subthemes related to the difficulties of navigating visitation restrictions when residents and family members were unintentionally afforded different levels of contact than others. In some facilities, this was due to characteristics of the physical environment, such as the variations between rooms or floors of the building, or in terms of access. For example, residents located on the ground floor of a building were able to see visitors through windows, whereas those on higher floors were unable to.

“*So the was bickering between sort of the residents that was aware of that and they were like ‘well, it’s alright for you, you can see your daughter through your window’ and all, and we were like ‘Oh, we’ll put the video on for you, (resident)’ You know? And they were like, well, it’s not fair my daughter can’t come and see me*.”

P008, F, Housekeeper

In other cases, this could be differences between LTCFs within the same locality and their approach to implementing restrictions. National guidance issued to LTCFs allowed for managers to develop an approach to visitation based on the characteristics of the facility and local conditions, such as infection rates, allowing the exact approach to such restrictions to vary between facilities. Once local lockdowns (restrictions limited to one geographical region) were introduced through the tier system, it was also possible that residents and family members could be residing in areas with different levels of restrictions. In some cases, this led to some family members moving residents from a facility with a relatively strict approach to visitation, to LTCFs with fewer restrictions, allowing them to visit.

“*Well, that lady, she moved her dad—fine. She was saying I’ll move him, I’ll move him. I said, well, if that’s what you want to do, that’s what you want to do, you know? But any home that’s going to let you go in and visit him, to me, it’s, you know, get on with it then, you haven’t got your dad’s best interests at heart, because surely keeping him safe should be your priority.*”

P002, F, Registered manager

The second subtheme related to inconsistencies in the approach to infection control and prevention strategies within the facility, in the context of wider restrictions relaxing outside the facility. Staff also questioned the government approach to visitation in LTCFs within the wider guidance issued to the general public. There was a sense of guilt reported by some participants that restrictions in place for residents did not apply to LTCF staff. At certain time periods during the pandemic, restrictions on family visitation were still in place while restrictions on the wider public had been reduced. During these time periods, LTCF staff were able to socialise outside of work, while residents remained isolated, leading to resentment from residents and staff members. In addition, given the potential for COVID-19 transmission among staff outside of the LTCF, restricted family visitation for the purpose of minimising the risk of COVID-19 transmission to residents was viewed as somewhat counterintuitive.

“*I think when it did start to ease a little but then it didn’t in care homes, that’s when it became even more challenging because you had staff that were then going off with COVID because they’ve been socialising together at weekends, well we can’t, you know, tell people what to do at weekends because they’re in their own time, quite rightly, but that really impacted on our residents because they were like, well how is that fair that they get to go out and I can’t go anywhere.*”

P018, F, Head of operations

Another participant reflected on the unfairness of continuing visitation restrictions as the pandemic progressed, during which restrictions were still in place within LTCFs as wider restrictions were removed. This created a double standard for staff members when inside and outside of the facility, which Participant P020 felt was unfair.

“*Well, I don’t know, it’s strange because we could go shopping, we could do everything and then we could come here, so it… I just find it strange that they couldn’t have the families come, you know, they could have had immediate family like, the mum, the daughter, whatever and come and see them, but they won’t let them. Government guidelines. But Boris could have a party*.”

P020, F, Senior care assistant

There was also criticism over the changes to visitation restrictions over the course of the pandemic, and the differences in approach for residents who died prior to the restrictions being replaced. In some cases, the strategies used to facilitate safe visiting, such as specific visitation rooms and outdoor areas, would have been available from the beginning of the pandemic, if allowed.

## 4. Discussion

This paper aimed to explore the experience of LTCF staff members in maintaining contact with families, including facilitating visitation and using remote technology, in England during the COVID-19 pandemic. In doing so, five themes were identified, each of which raise important questions regarding both the feasibility of implementing visitation restrictions and the extent to which the relative benefits of such restrictions outweigh secondary effects on residents, family members and LTCF staff. The findings add value to wider discussions on what an appropriate and proportionate response to managing pandemics in LTCFs could look like, and in doing so poses three questions that warrant further discussion.

The first question relates to the relative contribution of visitation restrictions on reducing the spread of COVID-19 in LTCFs. In a simulated comparison of the impacts of cohorting versus visitation on the transmission dynamics of COVID-19 in LTCFs, relaxing visitation policies did not significantly impact the cumulative number of infected residents, and variation in such restrictions had little impact on the probability of an outbreak in the facility within the first 90 days of the simulated epidemic [30]. In comparison, cohorting residents had a disruptive effect on the spread of infection, reducing outbreak size. In a Dutch study which explored the impact of permitting nursing homes to vary local protocols on visitation, subsequently increasing the possibilities for allowing visitors in the facility, there was a positive impact on residents and relatives without increasing the number of new infections [31]. As stated by one participant in this study, the purpose of restrictions on family visitation when there are few restrictions on staff members socialising outside of work is somewhat counterintuitive. Staff are already potential sources of infection, and this risk is elevated in staff members who work across multiple settings or who are asymptomatic [32]. The findings are in no way implying that restricting visitation is ineffective or unnecessary; however, its value in reducing the number and spread of infections within the context of wider policies requires further thought.

The second question relates to the extent to which successfully restricting visitation is achievable. The findings highlighted multiple challenges in implementing the guidance, related to the physical limitations of long-term care settings, i.e., multi-story buildings, LTCF staff lacking the time and resources to support visitation and difficulties enforcing restrictions with family members. Previous studies have echoed the additional difficulties experienced, for example in setting up isolation areas, cohorting staff and residents, and managing limited resources across quarantine zones [33,34]. Smaller-scale, home-like models of care have been associated with better quality of life for residents when compared to larger institutional settings, due to promoting resident autonomy and higher quality relationships, and have the potential to solve some of the physical issues which emerged during COVID-19 [35].

The findings from this paper show that restrictions on visitation are a sliding scale, both in terms of the policy itself and its implementation. In practice, the LTCFs included in this study were either limited in their ability to apply restrictions or were forced to bend the rules to allow contact beyond the scope of what was outlined in the policy guidance. In some cases, these exceptions led to increased visitation; in others, it led to unnecessary limitations. One example of this would be in the approach adopted to visitation at end of life; despite visitation under such conditions being allowed, one survey found that only 78.5% of facilities in the UK allowed families to visit residents who were near the end of life [36]. In terms of remote contacting strategies, these findings echo wider international research that identified a lack of emotional value in such approaches, alongside technological and human-related barriers to accessing these methods. In previous research, visitation strategies were highly valued when they allowed for emotional connection; in-person interactions were perceived to be more valuable than remote interactions, such as virtual visits, pre-recorded audio and video messages, and printed emails; however, the latter were rated higher in terms of feasibility [37].

The third question focuses on equity, proportionality and the secondary impacts of such policies. Inconsistency in the application of visitation restrictions, both between residents due to the limitations previously discussed, and between LTCFs that either interpreted the guidance differently or that developed visitation policies informed by individual risk assessments, led to inequality in the level of contact allowed between some relatives and family members. While the freedom afforded to LTCFs to prepare their own visitation plans allowed for approaches to be tailored to local needs, it also made LTCF managers vulnerable to potential criticism for their choices on this issue. In addition, the onus on LTCF managers to make these decisions increased the risk of a postcode lottery among LTCFs and the visitation restrictions they enforced [9].

### 4.1. Strengths and Limitations

To the authors’ knowledge, this is the first retrospective study to be conducted in LTCFs in England which takes into account the full timeline of visitation restrictions. In doing so, the findings can be contextualised within the wider experience of the pandemic, and this study allowed staff members to reflect on the changing approaches to visitation that were issued over the course of the pandemic. In addition, the inclusion of a variety of staff members, including managers, nurses and care staff, allowed for a multitude of perspectives to be collected, both from those making the decisions regarding visitation and those implementing them.

However, the findings are limited to one region in the UK, and to a sample size of twenty-four. In addition, this study excluded LTCFs which were rated as inadequate by the CQC; therefore, the experiences of LTCFs with the lowest rated quality of care are arguably absent from the data collected. In addition, there is a risk of recall bias due to the data being collected over a year after the final restrictions were removed. This is a potential limitation of this study; however, the extent to which the experiences of LTCF staff have been thoroughly explored is debatable, given the limitations of conducting research in such settings during the pandemic. In addition, this study does not capture the experiences of agency staff or those who have subsequently left the care sector. On this basis, the findings of this paper make a valuable contribution to a wider field of literature on the impact of the COVID-19 pandemic. This is especially so for LTCF staff, many of whom felt that their voice had remained largely unheard or forgotten since the pandemic ended.

### 4.2. Implications for Research, Policy and Practice

The findings of this paper have shown there is a knowledge gap in terms of both what is important for family members during periods of restricted visitation, and what is realistically achievable for LTCF staff to deliver. The appropriateness of visitation strategies will depend not only on the physical and cognitive capacities of the resident, but also on the needs of the family members, both of which may change over time. Previous research has identified that the more frequent family visits have been associated with improved resident quality of life, and combined with effective communication and relationship building with LTCF staff can improve quality of care [38]. During the pandemic, higher satisfaction among relatives was linked to having multiple opportunities to stay in contact with residents and the development of strategies within the facility such as the use of timetables or appointing a staff member as the primary contact [39]. Further research is needed to explore what is important to residents and relatives in terms of remaining in contact during periods of lockdown, and how these preferences can be delivered by LTCFs.

The COVID-19 pandemic led to a wealth of innovation from LTCF staff in creating spaces for visitation, some of which have been maintained as restrictions have been removed. One area for further research is the refinement and dissemination of such approaches, to ensure that this knowledge is not lost and can be adopted by or adapted to fit into other facilities. The potential for voluntary staff confinement, for example, whereby staff voluntarily confine themselves in the facility with their residents to reduce the risk of COVID-19 transmission, gained media attention in the UK, but evaluations of its effectiveness and the factors which affect staff willingness and acceptability are relatively sparse, despite promising results [40].

The findings in this paper also highlight the need to establish clear, consistent communication for relatives, both collective and personalised. Firstly, LTCFs and all stakeholders involved should benefit from the widespread advances in remote working which have become standard in other settings since the onset of the pandemic. This includes LTCFs having access to available technology and established procedures for using such resources, ensuring family members have access to social media or are supported in accessing it, and embedding remote contact with family members as standard practice prior to restrictions being adopted. These developments should include safety measures in place to protect staff from using their own personal phones or social media accounts to engage in contact with families. In addition, there is a need for established methods of communication for issued government guidance, ensuring that any guidance is clear, consistent and available in an accessible format for residents, family members and staff.

Based on the findings of this paper, there are five recommendations for future policy development. Firstly, to create consistent, accessible guidance that can be shared between all stakeholders, with clear routes of communication for updates at short notice. This would reduce the burden of responsibility on LTCF managers in communicating guidance to family members. Secondly, to support LTCFs in developing a variety of approaches to maintaining contact with family members and facilitating visitation which can be delivered from the onset of a local or national lockdown. The strategies implemented must be accessible to all residents, regardless of physical or cognitive capacity. Thirdly, to develop guidelines for the use of social media by LTCFs, establishing and embedding these approaches into routine practice prior to the onset of restricted visitation. Fourthly, to ensure guidance for visitation in LTCFs is complimentary to both co-existing policies within long-term care settings, and policies that have implications for LTCF staff members and their families, such as social distancing restrictions, that impact on the likelihood of being exposed to infections outside of working/visitation.

Finally, to address wider challenges related to staff shortages within the long-term care sector, to ensure an appropriate level of staffing is available to facilitate restricted visitation and support remote contacting. The impact of restricted visitation will undoubtably be one of the central areas of focus for the upcoming module five of the COVID-19 Inquiry, which will investigate the impact of the pandemic on the adult social care sector, and further work is needed to ensure lessons are learned for the future.

## 5. Conclusions

This study provides useful insights into the experiences of LTCF staff members in communicating and implementing visitor restrictions across the life course of the COVID-19 pandemic. The experiences of LTCFs during this time, and the lessons that can be learned from them to inform future pandemic preparedness, remain an important area of interest, both from a policy and research perspective.

## Figures and Tables

**Table 1:** Demographic and occupational characteristics of LTCF staff interviewed (n = 24).

Variables	Mean	(Range)	N	%
Age	45.9	28–68		
Gender—female			21	87.5
Ethnicity—white British			21	87.5
English first language			22	91.7
Years working in direct patient care	21.3	5–46		
Years working in care homes	18.2	5–46		
Full-time staff			19	79.2
Number of residents being provided direct care (2)	39.9	12–118		
**Current position or role**				
Registered care home manager			5	20.8
Deputy care home manager			2	8.3
Head of operations			1	4.2
Clinical manager			3	12.5
Senior care assistants/CHAPS team leader (1)			6	25.0
Care assistant			3	12.5
Activity co-ordinator			1	4.2
Housekeepers			3	12.5
**Highest level of education completed**				
Tertiary level (higher vocational training or university)			10	41.7
Higher-secondary level (up to A level or equivalent)			6	25.0
Lower-secondary (up to GCSE or equivalent)			8	33.3

(1) CHAPs—Care home assistant practitioners. (2) The mean number of residents each interview participant provided care to on a daily basis.

## Data Availability

All data generated or analysed during this study are available from the authors on reasonable request.

## Abbreviations

CQC	Care Quality Commission
CHAP	Care Home Assistant Practitioner
IPC	Infection prevention and control
LTCF	Long-term care facility
